# (Re)thinking about self-harm and autism: Findings from an online qualitative study on self-harm in autistic adults

**DOI:** 10.1177/13623613241271931

**Published:** 2024-09-04

**Authors:** Sarah JE Marsden, Rachael Eastham, Alexandra Kaley

**Affiliations:** 1 Lancaster University, UK; 2 University of Essex, UK

**Keywords:** autism, neurodiversity, online qualitative methodology, self-harm, self-injurious behaviours

## Abstract

Autistic people experience a higher prevalence of self-harming behaviours than do the general population. Self-harm remains a stigmatised topic, and until recently, self-harming behaviours in autism were considered to be limited to self-injurious behaviours experienced by intellectually impaired autistic children and not to be experienced by cognitively able autistic adults. Because of the belief held by many professionals that many mental health-related problems are inherently part of autism and immune to treatment or help, many autistic people are left unable to access the help and support that they desperately need. This study adopts an online qualitative methodology to explore this under-researched phenomenon, thematically analysing online forum posts from autistic adults to determine what forms of self-harm are described, what precipitates the self-harm and how forum users support each other. The findings reveal that self-harming behaviours are nuanced and complex, highlighting the connections between sensory overload, meltdowns, stimming and impulsive repetitive blunt trauma seen as autistic self-injurious behaviours. When seeking help, barriers include navigating misdiagnoses of other mental illnesses and being misunderstood or disbelieved by professionals. Online forum users offer help in the form of emotional empathy, as well as practical solutions, providing an example of valuable peer support.

**Lay abstract:**

There is a higher prevalence of self-harming behaviours within the autistic community than is experienced by the general population, in addition to co-occurring mental health conditions such as anxiety and depression. To date, research has focused on quantifying and categorising the numbers of autistic people self-harming, what types of harming they are engaging in and what functions the harming performs. Autism research has historically focused on the opinions and experiences of parents, carers and clinicians, with a belief that autistic people are unable to present their own experiences and thoughts accurately. This study adopted a qualitative method to develop themes arising from online forum discussions, using the words of autistic adults talking about how they experience and understand their self-harming behaviours. The analysis discovered that self-harming behaviours are used by autistic people as a way of coping with anxiety and depression and to relieve the build up of stress and sensory or mental overload that can otherwise lead to a meltdown. Repetitive behaviours such as stimming are also used to relieve the buildup of sensory over-stimulation and anxiety, but both stimming and meltdowns can also be self-harming behaviours if they cause tissue damage, and are believed to be childhood presentations which are stigmatised if expressed by an autistic adult. Many autistic adults find it hard to get help with self-harming behaviours because they are not taken seriously by professionals, as it is seen as part of autism and cannot be helped, or the professionals do not have enough knowledge of autism.

## Introduction

Autism is included within the American Psychiatric Association’s (APA, 2013) Diagnostic and Statistical Manual of Mental Disorders (*DSM*). This stigmatises autism as a mental illness and attaches a negative disorder label to what is essentially just a cognitive difference – a different style of processing sensory input and interacting with the world around us. To reinforce the negative association between autism and mental ill health, research has shown that there are comorbidities linked to autism and mental illness, the most prevalent being depression and anxiety (Crane et al., 2019; Hollocks et al., 2019). Understandably, there is value for autistic people in being accurately diagnosed with comorbidities that may help improve their quality of life (Lai & Baron-Cohen, 2015). However, this tendency towards diagnosis can also reinforce the negative, pathologising the association between autism and mental health.

Autism is identified as having two main categories of shared diagnostic similarities: difficulties in social and communicative interactions and a need for repetition and routine (Centres for Disease Control and Prevention, 2024; National Autistic Society (NAS), 2024). These shared similarities can all contribute to raised anxiety levels, inducing physical responses that are stigmatised by society. Self-stimulatory behaviours, or stimming, are repetitive physical movements that are believed to dissipate anxiety before it reaches a critical pressure point (Kapp et al., 2019). If a person is unable to release this pressure, meltdowns can be an intense mental and physical response (Belek, 2019), losing control to the point where a recovery period is needed, and shame is felt. Depression, self-harm and suicidal ideation can potentially occur as a consequence of experiencing overload, anxiety and taboo behaviours. The knowledge of having these differences and having to conform to a society and environment created by and for a predominantly neurotypical population can create a sense of thwarted belongingness, isolation, burdensomeness and inadequacy (Cage et al., 2018; Moseley et al., 2022).

Self-harm in the autistic community has been historically framed as something that occurs as part of the repetitive and restricted self-injurious behaviours (SIBs) observed in intellectually impaired children (Minshawi et al., 2014), and not something that is also experienced in cognitively able autistic adults. Self-harm in the general population has been historically linked to borderline personality disorder (Chandler et al., 2011), but because of its prevalence in the general population without co-occurring mental illness diagnoses, non-suicidal self-injury (NSSI) disorder is now separately recognised within the APA’s Diagnostic and Statistical Manual of mental disorders (5th ed.; *DSM*-V; Zetterqvist, 2015).

The few studies that exist on self-harming behaviours in the cognitively able adult autistic population all confirm an increased prevalence in the autistic population in comparison to the general or control population (Cassidy et al., 2018; Gilmore et al., 2021; Goldfarb et al., 2021; Maddox et al., 2017; Moseley et al., 2019, 2020; Steenfeldt-Kristensen et al., 2020). Maddox et al. (2017) found that there was no significant difference in terms of form and function between self-harming behaviours in autistic adults and those of the general population but hypothesised that this gets overlooked because of the occurrence of SIBs in autism. Moseley et al. (2019) validated and extended upon these findings by adding a qualitative component to their survey and reported that alexithymia, difficulty identifying and expressing emotions, was significantly associated with self-harm as a function of regulating high-energy states. Moseley et al. (2020) found a positive correlation between suicidality and self-harming incidence and that cutting as a mode of harming was a significant predictor of suicidality. As the current sole qualitative study on self-harming behaviours in autistic adults, Goldfarb et al. (2021) portrayed self-harm as being a more compulsive, uncontrollable and autism-specific phenomenon located within the wider context of repetitive and restrictive behaviours (RRBs). Blunt trauma such as self-hitting or crushing pressure is used in response to sensory or emotional overload or to self-stimulate because of altered sensation, but the study population of Goldfarb et al. (2021) all function well enough to partake in qualitative interviews, so self-harm here crosses the divide between SIBs and NSSI. They acknowledge the limitations of their small sample and suggest that as RRBs are so prominent in the autistic population, a larger study is warranted. They go on to discuss the taboo nature of the topic, which may not be shared within either research or clinical investigations, and also the need for reflection within autistic people that is made difficult during face-to-face interviews.

This study addresses these shortcomings, as a larger population is accessed via online forums, and the forums also provide an anonymous place for people to present personal representations that have been written without time pressure.

Autistic adults without an intellectual disability can go undiagnosed until later in life when often a stressful life event precipitates the need to seek help from professionals (Jones et al., 2014; Lai & Baron-Cohen, 2015). Because they have had to navigate life previously masquerading as neurotypical, autistic adults can find it hard to be believed by others because they do not present outwardly as autistic (Davidson & Henderson, 2010). This lack of understanding is also mirrored by limited availability of services for this specific sub-population, and tendencies to misdiagnose within mainstream services (Jones et al., 2014; Lai & Baron-Cohen, 2015). The neoliberal context of the United Kingdom means that responsibility for any labelled mental disorders, including autism and self-harm, is placed directly upon the individual. This represents a form of systemic oppression at the level of the individual; therefore, typically the contributions made by wider society to these conditions are overlooked (Inckle, 2020).

With evidence suggesting a preference for written communication, online autistic communities function as a safe space for many autistic adults to access information, advice and support (Brownlow & O’Dell, 2002, 2006; Jordan, 2010). Community support for autistic people via online forums is advantageous for a myriad of reasons including anonymity, accessibility, likeminded people experiencing similar situations and the avoidance of face-to-face interactions (Benford & Standen, 2009; Jordan, 2010). The safety afforded via the anonymity and autonomy of participation in online forums means that conversations held in this space may represent the feelings and experiences of autistic people, and this meant it was an appropriate focus for this research. Understanding self-harm from the perspective of autistic people allows us to draw out the differences and commonalities with non-autistic self-harm (a topic more widely studied). This presents valuable opportunities to understand needs and potential support options. Most importantly, this ensures that the voices of autistic people lead to understanding about autistic self-harm. This study also demonstrates the unique insights available through online forums where members chat freely, led by their own interests and experiences.

The aim of this study was to gain an understanding of the relationship between autism and self-harm, from the perspectives of adults with intact verbal ability allowing them to self-express in writing in online forums. This was reflected in the overarching research question ‘How is self-harm experienced and shared within the online autism community?’ Supporting research questions were:

1. What reasons do autistic people give for their self-harm in online forums?
2. What are the forms of self-harm described by autistic people?
3. Are there any perceived barriers to seeking professional help for self-harm in autism?
4. How does the online autistic community support and respond to descriptions of self-harm?

## Method

In order to uncover the subtleties of the subjective experience of autistic self-harm in an uncensored way, without causing undue burden to participants, an online qualitative approach was adopted. An online methodology allows access to a wider population and a larger sample than with traditional qualitative research, as those who are geographically isolated or physically housebound are able to connect with others within the virtual space (Giles, 2017; Wilkinson & Thelwall, 2011). Using online interactions qualitatively allows a unique level of access to an otherwise hard-to-reach population, as the social and communicative differences that members of the autistic community experience can limit their active participation in conventional qualitative research (Botha et al., 2021). Within the context of researching sensitive topics such as self-harm, an online methodology has a distinct advantage over conventional qualitative methods. It can be argued that interviews or focus groups create fresh distress by recalling memories or emotions, and the presence of a researcher or the nature of the questions asked may limit the truthfulness or extent of responses (McDermott et al., 2013). When recruiting for traditional qualitative methods, participants are limited to those who are comfortable talking to researchers or strangers, and in the case of sensitive topics, those who feel able to open up about a personal subject (McDermott et al., 2013; Wilkerson et al., 2014). Self-harm is often hidden and may not be brought to medical attention, so recruitment by traditional means may end up excluding the majority of the population of self-harmers (McDermott, 2015).

### Sampling strategy

Two contrasting but prominent autism online forums were selected for data collection. The first forum accessed was a charity in the United Kingdom, created initially by parents of autistic children to provide support and a point of contact for information, but with an active community of autistic adults. The second forum accessed was a public forum with no charitable affiliations, based in the United States with global contributions. A purposive sampling strategy was used as data was collected specifically from online spaces dedicated to autism, and within these, only from those who were discussing aspects of self-harm. The sample size of the data was limited by both available data and the decision that the research questions had been adequately answered by the richness of data collected. Autistic adults and young adults were the target population, and contributors to both communities were restricted to those over 16 years of age. The necessary requirement of access to technology and the ability to communicate by written word limited the contributors to those with the intact ability to self-express in writing but could also include those considered to be minimally verbal.

### Data collection

The data collected was in the form of threads containing a whole discussion involving multiple participants, copied into a word-processing document ready for analysis, and deidentified and anonymised at this point prior to importation into NVivo12. Both forums had a search bar that enabled terms to be searched for within the threads, and the terms ‘self-harm’, ‘self-injury’ and ‘hurt myself’ were initially used to generate relevant material. The use of medical or research-oriented terminology such as ‘non-suicidal self-injury’ was not appropriate here, as the forum contributors use lay language only. After searching for the first three terms, the content indicated that much of what the participants considered as self-harm was actually in the context of meltdowns and self-stimulatory behaviours, or stimming; so search terms ‘meltdown’ and ‘stimming’ were also used. All searches were ordered for relevance, which diminished with each new page, until pages 10 and above held nothing new of any relevance. A difference in language used within the international forum created a new search term ‘harmful stim’, which was then used to search within the UK forum and revealed two new threads not found with the previous terms. Any threads concerning children were rejected, as self-reported adult experiences were the focus of the study.

This study did not work directly with participants. The only demographic characteristic relevant in this study was that the person was autistic. While this experience is undoubtedly mediated by different demographic factors such as gender, age and location (and the related discrimination and inequities); demographic information was not available in the online forum spaces in a way that would allow for meaningful extraction and analysis.

### Data analysis

Data was analysed using the reflexive thematic analysis method as described by Braun and Clarke (2006, 2019). Data was initially read on collection and was subsequently re-read on multiple occasions to enable familiarity. The data was then considered on three levels – as single statements, part of the whole conversation thread and within the context of the wider dataset. Hand-written notes were made upon reading each thread, including the researcher’s thoughts and feelings, before coding was undertaken within NVivo. Theoretical literature was considered during coding to aid in the organisation and mapping of the data. The coding was initially data-driven and inductive, but it became apparent that threads were selected according to their relevance to the research questions. As coding followed the themes of the research questions, it became deductive. Each dataset was revisited following initial coding, and more text was coded in subsequent rounds. Only one new code was created from the international dataset, and the two datasets supported each other well in terms of experiential richness. Themes were identified as answering the research questions, and sub-themes developed within these three themes. The themes were reviewed by returning to the codes and were also considered within the context of the whole dataset and the research questions. The themes were finalised during the writing of the findings and linked to the wider literature and theory during the discussion.

Data analysis was undertaken by the first author due to the necessity of it being a doctoral research project, but the whole process was validated at every stage by two other researchers. A reflexive journal was maintained throughout as an essential component of this qualitative methodology.

### Ethics

Guidance was sought from both the British Psychological Society (BPS; Hewson et al., 2017) and the Association of Internet Researchers (AoIR; Franzke et al., 2020) regarding specific concerns raised by the use of publicly available internet content. Forum posts were only collected from publicly available discussions, and both websites contained clear information to contributors regarding the public nature of any material posted. Where possible, the website owners were contacted to obtain permission to use relevant material from the forums. The anonymity of individuals and their posts was preserved by the use of alternative identifiers and by the removal of any identifying features during data processing. Verbatim quotes were not used when reporting the findings so that these cannot be entered into a search engine and traced back to the source. Ethical approval for this study was obtained from the Lancaster University Faculty of Health & Medicine Research Ethics Committee.

Community members were not involved in the creation of the study as it was a PhD project, but the student researcher identifies as part of the autistic community. The student researcher created the project, collected and analysed the data and produced a thesis for examination by viva.

### Reflexivity

The decision to take a qualitative approach was influenced partly by the researcher’s philosophical alignment with critical autism studies and the neurodiversity movement, which state a need for inclusive research and representation of the lived experience of autistic people in a predominantly non-autistic society. The selection of an online non-participatory methodology was justified by the limitations imposed upon face-to-face qualitative research at the onset of the Coronavirus pandemic but was also in sympathy with the researcher’s own heightened discomfort induced by the additional communicative pressures of face-to-face interactions and the associated eye contact and body language interpretation distractions. The student researcher has lived experience of autism, and so regardless of the autistic forum discussions being weighted more towards the autistic SIBs, they may have been subconsciously drawn towards those accounts that resonated with their own experiences.

## Findings

To preserve the anonymity of the forum participants, verbatim quotations were not used to illustrate direct examples within the themes. To distinguish between author interpretations and participant experiences, all materials derived directly from participants are demonstrated instead using *italicised text*.

The noun allistic is used interchangeably with non-autistic to refer to people who are not considered to be autistic.

## Theme 1 – ‘it ain’t what you do, it’s the way that you do it’ – making sense of self-harm

The first theme presents reasons why those on the autism spectrum self-harm and the forms of self-harm that they describe. This is divided into two sub-themes: self-harming behaviours considered to be part of being autistic and more conventional non-autistic self-harm as experienced within the general population.

### Sub-theme – self-harm as part of autism

Much of the self-harm described on the forums can be attributed directly to autistic traits and is essentially about management of information, whether it be too much or not enough. One of the cardinal criteria for an autism diagnosis and experienced by all on the spectrum to some extent is altered sensory perception, in the form of some senses being heightened and others diminished in comparison to those considered non-autistic (Pellicano, 2013). Cognitive processing can also take longer, so the brain and senses can quickly become overloaded, manifesting in a buildup of tension, which needs to be released in the form of a meltdown (Belek, 2019). If not released, it may be channelled into management techniques such as self-harm or stimming. Stimming presents a more compulsive, repetitive, unconscious way of dissipating an accumulation of anxiety and negative energy, which can also be destructive if it involves physical harm.

Overload or meltdown features strongly in reasons given for acts of self-harm, *with participants describing no specific trigger every time, just a buildup of multiple smaller things until it becomes too much to process. Meltdowns are described as a loss of physical and mental control, lashing out at others, inanimate objects or the self. One participant refers to it as a blackout, as if they experienced a total loss of consciousness, returning to reality with no recollection of what happened*. Self-harm here is unpremeditated, and *participants mention a compulsive urge to hurt themselves as part of the meltdown even if they know it is wrong, a reflex behaviour, which can also be hard to stop until the pain becomes too intense. The pain created shuts off the overloaded channels within the brain by creating a new overriding physical sensation to process, like a reset switch – participants liken the meltdown to blowing a fuse in the brain, flipping a switch or a circuit-breaker*. Hitting is the most prevalent form of self-harm during overload events, revealed by participants as *hitting the self on blunt objects repeatedly, or hitting themself with their hands, most commonly targeting the head, sometimes until bruised or teeth are damaged*. This can also become worse over time, with *some relating a progression from hitting objects to hitting the self and using more visible areas such as the head and face. Biting is also mentioned by a few participants, most often the hands, sometimes until blood is drawn or visible toothmarks are left as a reminder.*

*Public displays of these harmful meltdowns are common*, highlighting this difference between autistic and non-autistic forms of self-harm, *although some mention an ability to control the urge to meltdown until alone and in private. More commonly, anecdotes include members of the family who may have triggered the overload, having to witness, restrain, or even sustain injury from the ensuing physical release of stress. Restraint is noted as always making the situation worse, as it generates more sensory over-stimulation.* Family members are also not the most tactful here, sometimes mocking the individual due to misunderstanding. *One participant reports being filmed during the meltdown and having it replayed back to them afterwards to show them how it appears to onlookers, as an attempt to shame them into stopping the behaviour. Some self-harm during a meltdown is justified as a redirection of aggression towards others, turning the anger in on themselves in order to avoid hurting others*, suggesting a certain level of consciousness around the event. *Others describe turning it upon themselves in an effort to avoid the full-on meltdown, cutting it off before it escalates, so the experience of uncontrolled meltdown is considered to be worse than the more controlled act of self-harm*. It is often in these cases that cutting and burning, as more ‘conventional’ methods of self-harm, are used.

Stimming is another autism-specific behaviour that is mentioned significantly within threads on the topic of self-harm. Self-stimulatory behaviour, or stimming/stims as it is described by the autistic community (Charlton et al., 2021), is another unconscious physical repetitive way of reducing the buildup of anxiety and stress before it reaches meltdown levels. *Biting the lips, or inside the mouth, as well as biting skin on fingers or nails, is commonly described, as well as generally picking the skin. Skin picking is either done in private locations on the body, where it cannot be viewed by others, such as the scalp under the hair, or the feet; or is entirely visible, involving the face and hands, sometimes to the point where no nails remain and scars are left. Re-opening of scabs is described as a compulsion, worrying at something that does not feel right until it has been removed, reducing the accompanying anxiety. The sensations of bleeding and pain create a sensory overload that can be useful if a meltdown is building up, as these new sensations can function as an override switch. One participant justifies their scalp-picking as a localised way of shutting off more distant sensory stimulation coming from the rest of the body. Once in this pattern of stimming, it is felt by some to be comforting or soothing*. This form of stimulation blurs into sensation-seeking, where altered sensory sensitivity is reduced rather than heightened, and *pain is described as bearable, even pleasurable, feeling something physically in order to feel present mentally or a way of connecting with the senses. One participant describes relaxation of the body when having skin picked by someone else, a pleasurable loss of control and feeling a connection with a loved one.*

### Sub-theme – allistic self-harm

The act of harming is often presented by participants as a way of processing emotional pain, a physical expression of overwhelming feelings or memories. Although alexithymia is only explicitly mentioned once, some describe classic symptoms of not being able to describe their feelings adequately. Physical methods provide an alternative mode of communication, including using the scars as a reminder. *One participant relates a time when they wanted to demonstrate to a loved one that they could empathise with their emotional pain by cutting and causing physical pain.*

*Self-punishment for not being ‘normal’, not conforming to society, not being good at being human, being a disappointment to relatives or getting in trouble with the authorities are all given as reasons for this more premeditated and controlled form of self-harm. Self-harm alongside suicide attempts is discussed in the context of low self-esteem and feelings of worthlessness, commonly in conjunction with narratives of childhood trauma or historical abuse from family members. One participant describes an overly-strict upbringing with daily beatings for minor misdoings, which created the habit for punishing themself with a variety of methods and feelings of worthlessness that led them to regularly contemplate suicide. Another reveals that unpredictable parents and sexual abuse from a relative left them a long-term self-harmer to cope with the memories, which led to multiple hospitalisations after cutting went too far. A third participant attributes their self-harm to feeling unable to self-advocate in situations of mental abuse from family members, turning it upon themselves as a way of coping when there was no one to help.*

Anxiety is always present at low levels and builds up with stressful experiences until it needs releasing in some way, either via a meltdown or by a more premeditated, controlled and private self-harm, usually in the form of cutting. *Participants describe the pain and adrenaline rush, as well as the sight and sensation of the blood providing a temporary relief from the constant background noise of anxiety. The knowledge that it may upset those close to them means that cutting is undertaken in private and most often in places on the body that are easily concealed, as it leaves scars.* In contrast to this, self-harming to relieve depression is related more to using the physical pain to exteriorise emotional pain. This may have a sensation-seeking aspect if using the pain of harming in order to feel something. Cutting as a form of self-harm is most frequently mentioned alongside mental health diagnoses and is also described as being *controlled, premeditated and private.* The term *‘conventional’ or ‘non-autism-related’ self-harm* is used by some to define and differentiate that from autistic self-harm. *One participant mentions having a first aid kit prepared in order to avoid the embarrassing trip to hospital, after accidentally cutting too deeply, and another agrees with not cutting too deeply and also states the importance of keeping the wounds clean afterwards.*

## Theme 2 – ‘running up that hill’ – barriers to seeking help

The second theme presents perceived barriers to seeking help for self-harm as an autistic person. This is divided into two sub-themes: The first describes misunderstandings by professionals and the associated misdiagnoses, and the second highlights communication issues and not being listened to.

### Sub-theme – being misunderstood by professionals

Negative experiences are related by forum posters when interacting with medical or social care professionals, mostly due to a lack of knowledge and resources. *Misdiagnoses are felt to be common, as participants interacting within these threads describe themselves as at the cognitively able end of the spectrum with less obvious characteristics and the ability to mask their autism in order to fit in with society.* Misdiagnoses or comorbid diagnoses mentioned within the threads are *borderline personality disorder/emotionally unstable personality disorder, post-traumatic stress disorder, attention-deficit hyperactivity disorder, sensory processing disorder and obsessive-compulsive disorder. Participants attribute their misdiagnoses to professionals having greater knowledge and experience of other related disorders, finding salience in particular symptoms from other conditions and seeming satisfied as long as their patient gets a diagnosis. Once a diagnosis is given, it is then much harder to return and contest this or ask for a second opinion. One participant describes visiting a psychiatrist who was experienced in psychosis, and so focused in on their auditory hallucinations, wrongly prescribing an antipsychotic medication. This was later revoked by a psychologist who re-diagnosed the participant as having autism with synaesthesia. One participant was incredulous when told by a professional that they could not be autistic because they did not look or behave like an autistic person, with rocking and hand-flapping used as the diagnostic criteria. Another was not taken seriously because they could correctly determine emotions when presented with a range of emojis.*

Even if an autism diagnosis is eventually given, this experience is often bad enough to ensure that when the autistic person begins to self-harm, they do not feel comfortable enough to return to mental health services and go through the distressing process again; which is why participants reach out for help via the online forums instead. *One participant describes being refused help for mental health and self-harm because they did not appear visibly distressed due to differences in communication and expression, and another got told they did not look depressed because they smiled on greeting. Where individuals attended Accident & Emergency, some relate negative experiences including feeling that they are wasting time that could be spent on ‘real’ patients. One participant was even made to believe that they had inadequately self-harmed, so they subsequently returned home and harmed themselves in a much more severe way.*

*Attendance at social meetings and therapy groups were prescribed for a participant experiencing low mood, but this increased their distress by having to go out and interact with strangers and overload their senses. Another participant mentions that help is available as a child but then abruptly stops on reaching 25, even though the autism does not. Multiple participants reveal having to educate professionals, including sending information to them about autism. Diagnosis is perceived to be an important part of getting help, as professionals seem to require written proof as evidence before they will make efforts to help autistic people with mental health issues.*

### Sub-theme – not being heard

Differences in communication style, and the requirement for more time to process information, may cause non-autistic family and friends to speak for the autistic person, either in a well-meaning but unhelpful way or in a more controlling and manipulative way. *Some participants contact the forum for outside opinions on whether they are being abused or controlled by family members, including being sent for professional help against their will, as well as being spoken for by family when meeting with professionals as if they were not there*, suggesting that the allistic perspective on their self-harm and autism is the only valid one. *Others describe being taken advantage of by others, which leads to low self-esteem and subsequent self-harm. One participant was treated badly by a social care professional and lodged an official complaint, but the professional’s account was believed over the autistic person’s word*, suggesting that an autistic person is perceived as having less worth in society. If family and professionals cannot be trusted, there is nowhere else to go, leaving a sense of helplessness, feeling trapped and alone, with self-harm being the only outlet. *Many participants mention feeling unable to stand up for themselves in confrontational situations with others, not knowing what to say or do. They attribute this directly to their autism, as their brains fill up with too much information and they end up withdrawing into silence and self-harm.*

## Theme 3 – ‘when the going gets tough’ – support from the online community

The third theme presents the ways in which the online autistic community responds to posts describing self-harm. This is divided into two sub-themes: emotional and practical support.

### Sub-theme – emotional support

Responders to posts on self-harm reply empathetically with *admissions of similar situations and openly relate their own experiences of harming* to help the person feel that they are not alone. *Empathy is offered rather than helpful strategies when self-harm is described as being an addiction, and also when understanding the relief that it can bring from overload. There is an acknowledgement that autistic people will not deal with situations in the same way as non-autistic people. A common complaint is that allistic people will never understand autistic people, so one participant reminds the community that this is a two-way thing, as autistic people will also never be able to see things from an allistic perspective and that friends and family will usually act with their best interests at heart, even though sometimes misguided. Much support comes from those stating that they are older and have had more time to come to terms with their autism, providing reassurance that others will love their autistic self more as they learn to accept their differences.* Reassurance of self-worth also features strongly within the threads, with *reminders that everyone is flawed, not just because they are autistic and that they should value their life and contributions made to society, however small.* There is a focus on strengths gained from being autistic here and on how these can be used in a positive way, including the ability to think logically and view situations from a unique perspective. Some advise channelling the self-control and focus required to self-harm into other less harmful behaviours or strategies.

### Sub-theme – practical support

Redirection of the urge to self-harm is focused upon activities such as *snapping an elastic band on the wrist or applying an ice cube or hot wax to substitute the pain. Immersion in computer games is one suggestion, to induce a flow-state of hyper-focus that calms the mind. For meltdowns and overload-induced self-harm, knowing what is happening and learning the triggers is important, so that the full meltdown can be averted, walking away and giving the self some time out. Common triggers cited for a meltdown are hunger, fatigue, sensory over-stimulation and unexpected change of plans; so being prepared in advance by taking snacks and naps can reduce the buildup of multiple triggers. Refocusing the senses is recommended using a favourite piece of music or nature sounds, a calming scent, eating a spicy snack or chewing gum, or carrying a smooth pebble or fidget spinner to occupy the hands. For reducing harmful stims, switching to a less-harmful stim such as rocking or hand-flapping is suggested, and practising this is encouraged so that it becomes an automatic behaviour when losing control in a meltdown. In the privacy of familiar surroundings, using soft furnishings to hit the self, or using weighted blankets to apply pressure and refocus the senses, can avoid the need to hurt the self when alone. One participant describes being told by a professional to buy objects specifically to be smashed in anger when feeling a meltdown coming on. Pets are non-judgemental, and stroking them is soothing; one person recommends having an autism-assistance dog which reduced anxiety and meltdowns significantly.*

When seeking professional help, those who describe being able to self-advocate and navigate the complex healthcare system are the most positive. *Specific advice includes to get the professional to always write everything down so that it can be presented to other professionals at future appointments, as this makes the path smoother. Knowledge of the legalities of support and disability is also advised, with more than one participant quoting that professionals have a ‘duty of care’ to their patients, that service users are legally customers and that sections of specific acts are being broken when an autistic person is refused care or treated badly by professionals.* If participants contact the online community because they feel they cannot self-advocate, *contacting specific charities or advocacy services is recommended by some, and a few even offer to advocate on behalf of the original poster if necessary.*

## Discussion

The self-harm discussed in the autism online forum threads is a combination of what could be described as non-autistic self-harm and autism-influenced SIBs, but what is important is that all the acts are described by the participants as self-harm, whether they conform to the stereotype or expected behaviour pattern. Much of the self-harm described within the forums is autism-specific but does not fall neatly inside the categorisation as described within psychiatry, so is under-researched and under-represented within the literature. Unpremeditated blunt repetitive trauma in response to sensory overload in the form of a meltdown is considered to be autistic self-injurious behaviour. SIBs are a form of self-harming believed to be only experienced by children with autism or autistic adults with intellectual impairment and not by autistic adults without intellectual impairment (Karim & Baines, 2016; Matson & Turygin, 2012). Prior to this study, only one single qualitative study considering self-harm in autistic adults without intellectual impairment suggested that SIBs could exist as self-harm within this population (Goldfarb et al., 2021).

Reasons for non-autistic self-harm still resonate and apply to autistic self-harm, regardless of the method used. Explanations presented in the sociological literature for self-harm include emotional pain being channelled into physical pain (Chandler, 2013; Edmonson et al., 2016; McAllister, 2003), a way to regain control from chaos (Chandler, 2013, 2014), a history of mental or physical abuse from family and others (Chandler, 2012; McAllister, 2003), self-punishment for not conforming to an ideal (Edmonson et al., 2016; McDermott & Roen, 2016b), not being taken seriously by professionals (Chandler et al., 2020; Harris, 2000), being marginalised by society (McAllister, 2003; McDermott & Roen, 2016a) and the pathologisation of social deviance (McDermott & Roen, 2016a), all of which feature within the online forums where autistic adults are communicating their difficulties. Participants describe self-harming as a physical release of sensory or emotional overload in the form of a meltdown, or as part of socially unacceptable self-stimulatory behaviours. These can develop into more conventional forms of self-harm if they also experience mental ill health or are made to feel abnormal by family or professionals. This can exacerbate the harming behaviour, as it becomes a form of self-punishment for not being ‘normal’, or for transforming the emotional pain into physical pain in order to process the experience.

The shame and stigma of having an uncontrollable autistic meltdown in public is palpable within the threads, with both close family members and qualified professionals treating the autistic person poorly during and after the event. Specific examples given include making them stop by restraint or ridiculing them afterwards in the hope that they will be shamed into not repeating the behaviour. These physical manifestations of mental overload and distress are coping mechanisms, often described as a last resort when there is no other escape, and self-harm emerges as a way of dealing with stress. Within the sociological self-harm literature, this is described as a ‘pressure-cooker’ situation, where self-harm is used as a release for an overload of emotions (Brossard & Steggals, 2020). This takes the affect-regulation theory of self-harm in psychology further by positing that pressure applied by society to appear or behave in a certain way creates the need to self-harm and release this pressure (Brossard & Steggals, 2020).

A disproportionate amount of autistic self-harm discussed within the forum is linked to stimming, and this is a novel finding in autistic self-harm research. These repetitive and compulsive behaviours are often performed unconsciously to alleviate the buildup of anxiety or overload. Often described as self-soothing, they provide a protective mechanism against distress even if considered by outsiders to be physically harmful. Linked mainly to autistic children in psychiatric literature, these behaviours are stigmatised if they are experienced as being exhibited by adults (Kapp et al., 2019), as they fall outside what is ‘normal’ or acceptable, creating minority stress. Minority stress is caused by the stigma of being ‘othered’, where both autistic people and self-harmers are made to feel inadequate and wrong in comparison to ‘normal’ society for their deviant behaviours (Botha & Frost, 2020; McDermott & Roen, 2016a). This reduces mental wellbeing, creating a vicious cycle of needing to self-harm in order to punish the self for being abnormal or alleviate the buildup of negative thoughts and emotions. Framed by the neurodiversity paradigm, this form of self-harm can be viewed in a more positive light, and there is an argument for such behaviours to be left alone if they serve a purpose (Leadbitter et al., 2021; Milton & Moon, 2012). Self-stimulatory unconscious behaviours such as hair twiddling or leg jiggling are considered acceptable within the non-autistic population, being neutrally attributed to restlessness or boredom, and are only viewed negatively when associated with autism (Pearson & Rose, 2021).

The intersection of being both autistic and self-harming, and the associated stigma of both of these marginalised identities, compounds any negative effect further, making it even more impossible to seek help or feel accepted by society. Many of the forum posts regarding mental health diagnoses also reflect an inability by professionals to take into consideration the overlap between autism and many symptoms of mental illness diagnoses. A mental illness diagnosis is often settled for over an autism diagnosis because of the siloed categorisation of disorders within the *DSM*-V and the associated monolithic thinking of psychiatry as a science.

Poor professional knowledge of autistic traits was a strong theme within the forum discussions, with some describing having to educate healthcare professionals about aspects of their identity or on the more subtle presentations. Others were told that they could not be autistic because they were not showing the more stereotyped behaviours associated with a diagnosis. Participants also felt that if they did not conform to the classic depiction of autism, they were dismissed, creating a barrier to seeking help. If an autistic person presents with subtle or ‘mild’ traits, masking or considered to be higher-functioning and capable of self-advocacy, there is a general misconception that the person does not need help (Gillespie-Lynch et al., 2017; Milton & Moon, 2012). Many of the forum participants admit to making autism their specialist subject and use knowledge to their advantage when negotiating with medical professionals, some being confident enough to offer advocacy on behalf of others. The use of ‘strategic medicalisation’ can be found within the wider literature of other marginalised communities, such as the trans community, when articulating medical needs or validating beliefs (Johnson, 2019).

The neurodiversity movement promotes active involvement in the medical sphere by self-education and advocacy, using medicalised terminology to obtain a diagnosis and legal knowledge to access support (Russell, 2020). This is augmented by the online community sharing information and experiences, with social identity theory underpinning the positive connections that are made by meeting others with similar differences (Tajfel & Turner, 2004). The forum conversations also provide direct support for Milton’s (2012) double-empathy theory, which argues against the cognitive theory of mind (Baron-Cohen et al., 1985) and later empathising systemising theory (Baron-Cohen, 2009). Both theories suggest that autistic people lack the ability to understand or imagine what other people may be thinking and therefore are unable to empathise with others. This study revealed that within the online autistic community, empathy was shown for both fellow autistic people and non-autistic people. Some also portray their disagreements with family and professionals over harming behaviours as a mutual misunderstanding, providing more support for the double-empathy theory.

### Limitations

Although this study accesses more participants than usually stated within qualitative research, it is still not considered to be generalisable to the autistic population for multiple reasons. The online nature of this study limited the participants to those who were not only computer literate but also that had access to technology, although online methods do allow access for those who may be geographically isolated or unable to travel to physical meetings (Wilkerson et al., 2014). The relative anonymity of creating an avatar online means that demographic information was variable and incomplete, so could not be used. There is also an argument to suggest that people are not their true self when contributing online (Brownlow & O’Dell, 2006), which has been refuted by more than one researcher who suggest that anonymity actually allows for greater disclosure (Hewson et al., 2017; McDermott et al., 2013; Wilkerson et al., 2014). Because of the inability to obtain individual consent for the use of forum posts, it was impossible to illustrate findings by using verbatim quotes, which is considered by some to be an essential component of a qualitative study (Franzke et al., 2020; Hewson et al., 2017). The researcher’s insider involvement as an autistic person will have influenced what was focused upon and how the findings were represented. Because of the historical aspect of the postings and anonymity online, participants were unable to be used to member-check the findings, but a reflexive journal was maintained throughout to record the researcher’s thoughts and decisions.

## Conclusion

The findings of this study present a more autistic experience of self-injurious behaviours such as harmful stimming and meltdown self-harm, which predominate the discussions, alongside more allistic forms of self-harm as experienced within the general population. Self-stimulatory behaviours are a form of autistic self-harm not previously elucidated within research, and the online methodology presented a novel way to represent autistic voices on a sensitive topic. Shame and stigma attached to autistic compulsive behaviours means that cognitively able autistic adults feel unable to undertake behaviours such as stimming in public. Instead, when they are overwhelmed, the pressure can increase to the point of a meltdown or is released in private as a more controlled form of self-harm. Differences in communication can mean that autistic people are unable to adequately self-advocate, can be spoken for by well-meaning others and feel disbelieved if appearing to function well due to masking behaviours. Seeking help can involve navigating a minefield of misdiagnoses, and support offered is often inappropriate or insensitive to autistic needs. The online adult autistic community offers a place where likeminded people can ask questions and validate their experiences, and where empathy and practical solutions are shared.

A paradigm shift has begun within autism research. We need to continue listening to and involving more autistic people, concentrating upon real social and environmental changes that can improve the lives of those already living with autism, increasing awareness and acceptance through education of professionals and the public, making diagnostic services more widely available and accessible and having appropriate post-diagnostic support. Autism needs to be removed from official diagnostic categories and manuals such as the Mental Health Act in the United Kingdom, or the APA’s *DSM*, in terms of being labelled a mental illness or disorder (NAS, 2019). We need to re-write the ‘disorder’ narrative to become a positive ‘difference’, located within a spectrum of neurodiversity that we all exist upon. It is not autistic people that need to become more ‘normal’; it is society and the wider environment that needs to adapt to accommodate this difference, and then autistic people will not appear to be so different after all. As one anonymous autistic person is quoted as saying, ‘We are fresh water fish in salt water. Put us in fresh water and we are fine. Put us in salt water and we struggle to survive’ (Baron-Cohen, 2017).
